# Now Is the Time to Bring a Common but Unpopular Noncommunicable Disease into Focus: Peripheral Arterial Disease Takes Limbs and Lives, but It Must Also Touch Our Hearts!

**DOI:** 10.3390/jcm11195737

**Published:** 2022-09-28

**Authors:** Christian-Alexander Behrendt, Frederik Peters, Ulrich Rother

**Affiliations:** 1 Department of Vascular and Endovascular Surgery, Asklepios Clinic Wandsbek, Asklepios Medical School, 22043 Hamburg, Germany; 2 Brandenburg Medical School Theodor Fontane, 16816 Neuruppin, Germany; 3 German Institute for Vascular Research, 10115 Berlin, Germany; 4 Hamburg Cancer Registry, 22083 Hamburg, Germany; 5 Department of Vascular Surgery, University Hospital Erlangen, Friedrich-Alexander University Erlangen-Nuremberg, 91054 Erlangen, Germany

We have all learned a great deal from the ongoing pandemic that has already taken more than five million lives in less than three years. Innovative vaccines were developed, practice guidelines on the management of COVID-19 were issued rapidly, and observational research improved significantly while using a broad variety of administrative and clinical registries to dive into global real-world practices. Humans are capable of extraordinary things if the motivators are strong enough, and it certainly helps if there are both emotions and public interest behind such motivators.

Very early on during the pandemic, the World Health Organization (WHO) and national authorities issued that certain risk factors exist which predict severe illness in patients infected with SARS-CoV-2. Interestingly, almost every risk factor on that list was also very common in patients with lower extremity peripheral arterial disease (PAD) and, more recently, the disease itself has been identified as a major independent prognostic factor for adverse outcomes [1]. This adds to earlier studies reporting that PAD patients had four times the risk of suffering from severe acute respiratory failure when they became infected with the seasonal flu while the vaccination rates were far lower than the recommendations from the WHO [2,3]. Beyond infection risks, undertreatment is a general issue in this target population regarding patient education and appropriate secondary prevention. Strikingly, a survey study recently revealed that one third of patients with PAD were not aware of their medical prescriptions, and almost 50% stated that they had not changed their lifestyle and health behaviors since the index diagnosis [4].

People affected by asymptomatic and symptomatic PAD will ultimately develop a broad range of complex comorbidities during their life. Hence, the earlier we start to optimize the risk profile and establish superior medical therapies and lifestyle changes in the affected patients, the better the life expectancy. Thereby, PAD is becoming an increasing burden for health care and economic systems. Almost 237 million people were affected worldwide in 2015, while demographic developments and medical improvements led to an increasing prevalence [5,6]. Moreover, most recent estimations based on observational data and population growth support the likelihood that an increase of more than 30% can be projected until 2045. Furthermore, according to the most recent numbers of the International Diabetes Federation, 537 million people worldwide have diabetes. Thereof, 50% will develop lower extremity PAD, which typically affects tibial and pedal arteries that are considerably challenging to treat [7].

In the current Special Issue, we aimed to collect high quality publications on epidemiology, inequalities, and outcomes. With 13 accepted submissions in total, this collection of papers emphasized that the vascular community has a strong interest in extending the still limited knowledge base on PAD. Aside from two review articles, eleven observational studies presented real-world evidence on patterns and trends in the incidence and prevalence of PAD, the utilization of treatment, the prognostic risk factors and comorbidities, the long-term outcomes, and sex disparities. Hopefully, the generated hypotheses will be assessed in future research projects and randomized controlled trials.

Endre Kolossvary, an internationally renowned expert and author of numerous papers on lower extremity amputation practice, prepared a narrative review to illuminate striking inequalities and an East–West divide concerning this important indicator of outcome quality in the comprehensive care of patients with PAD [8]. Thus, disparities do not only exist on a national level, but also regionally. Trenner et al. used DRG reimbursement data derived from the German statistical office to determine how practice patterns differed between federal states in Germany [9]. Closely related to the latter publication, Jacobi et al. and Keller et al. used data from a large epidemiological cohort study and administrative data to determine the association between periodontitis and diabetes, respectively, and PAD [10,11]. There is an increasing interest in the modifying role of chronic inflammation in cardiovascular disease development while both diabetes and periodontitis may serve as good examples for research projects.

The evidence-based revascularization strategy and its corresponding outcomes were addressed by two further publications. While Wijnand and colleagues retrospectively validated the new Global Limb Anatomic Staging System (GLASS) and reported a low inter-observer agreement for unconditioned scoring, the GermanVasc group addressed an ongoing international debate in the endovascular treatment of PAD. More than two years after Katsanos et al. reported that treatments with paclitaxel-coated devices were associated with excess mortality in the summary level trial data, we revealed that cohorts in randomized controlled trials (RCT) vs. observational data differed widely. Most strikingly, females treated above the knee may have benefited from paclitaxel-coated devices, while no differences were found in males. The results also highlighted the modifying role of pharmacological treatment, which raised the interesting question of whether both the choice of and adherence to pharmacological therapies may actually have a higher impact on long term outcomes than the choice of devices [12,13,14]. Further diving into the possible differences between sexes, Porras et al. determined the frequency of primary healthcare contacts preceding the diagnosis of lower extremity PAD. In their study, patients with PAD visited a general practitioner more often than a similar population without PAD, regardless of sex. Yet, the hypothesis that reported sex differences in the severity of PAD at diagnosis are explained by a delay in presentation to the general practitioner could not be confirmed [15]. As vascular surgeons, interventional radiologists, and angiologists, we must appreciate that the majority of the life course of our patients is guided by general practitioners. They are the most competent gatekeepers of patient-centered care, which further emphasizes the importance of interdisciplinary communication.

Dimech and colleagues provided valuable insights into the Maltese treatment reality. Therein, the obvious advantage of this observational study is its completeness. A single center provides health care to the entire Maltese population, while 670 vascular centers in Germany are involved in the complementary invasive treatment of patients with lower extremity PAD. Perhaps most interestingly, a reduction in major amputation rates was associated with an increase in revascularization rates [16]. Almost 18,000 km and ten time zones away, Hart et al. aimed to describe the situation in New Zealand. Strikingly, the authors found that females were more likely to die after the treatment of CLTI compared to men [17].

Taken together, the publications included in this Special Issue again remind us about the uncomfortable truth that PAD is a particularly prevalent and burdensome disease with limited treatment options and devastating long-term outcomes [18]. Despite the efforts that have already been made, most guideline recommendations are still based on expert consensus or lower levels of evidence [7]. We urgently need better data and better research methods to identify effective strategies to prevent, treat, and—one day—hopefully cure the disease and alleviate its lifestyle-limiting symptoms [19]. Moreover, research efforts need to account for regional and socio-economic disparities and finally prioritize females and other disadvantaged patient subgroups. As in other diseases, the complex decisions around topics such as antithrombotic therapy and invasive procedures need to be much more tailored to the specific patient’s profile. Recently developed risk scores and staging systems such as Wound, Ischemia, Foot Infection (WIFI), GLASS, or the OAC3-PAD bleeding score (https://score.germanvasc.de (accessed on 27 September 2022)) should be externally validated and further improved using population-based cohorts [12,20,21]. Several observational studies have highlighted the low rates of the best medical therapies and foremost lifestyle interventions, but also optimal pharmacological treatment. The underlying barriers and efficient ways to overcome them, however, are still unknown.

The current Special Issue will remain open for submissions as a Topical Collection, and we invite you to submit your valuable contributions.
